# Morbidity and mortality in homeless individuals, prisoners, sex workers, and individuals with substance use disorders in high-income countries: a systematic review and meta-analysis

**DOI:** 10.1016/S0140-6736(17)31869-X

**Published:** 2018-01-20

**Authors:** Robert W Aldridge, Alistair Story, Stephen W Hwang, Merete Nordentoft, Serena A Luchenski, Greg Hartwell, Emily J Tweed, Dan Lewer, Srinivasa Vittal Katikireddi, Andrew C Hayward

**Affiliations:** a Centre for Public Health Data Science, Institute of Health Informatics, University College London, London, UK; b The Farr Institute of Health Informatics Research, University College London, London, UK; c Institute of Epidemiology and Health Care, University College London, London, UK; d University College London NHS Foundation Trust, London, UK; e Centre for Urban Health Solutions, Li Ka Shing Knowledge Institute, St Michael's Hospital, Toronto, ON, Canada; f Mental Health Centre Copenhagen and Institute of Clinical Medicine, Faculty of Health and Medical Sciences, University of Copenhagen, Denmark; g Department of Social and Environmental Health Research, London School of Hygiene & Tropical Medicine, London, UK; h Medical Research Council/Scottish Government Chief Scientist Office Social and Public Health Sciences Unit, University of Glasgow, Glasgow, UK

## Abstract

**Background:**

Inclusion health focuses on people in extremely poor health due to poverty, marginalisation, and multimorbidity. We aimed to review morbidity and mortality data on four overlapping populations who experience considerable social exclusion: homeless populations, individuals with substance use disorders, sex workers, and imprisoned individuals.

**Methods:**

For this systematic review and meta-analysis, we searched MEDLINE, Embase, and the Cochrane Library for studies published between Jan 1, 2005, and Oct 1, 2015. We included only systematic reviews, meta-analyses, interventional studies, and observational studies that had morbidity and mortality outcomes, were published in English, from high-income countries, and were done in populations with a history of homelessness, imprisonment, sex work, or substance use disorder (excluding cannabis and alcohol use). Studies with only perinatal outcomes and studies of individuals with a specific health condition or those recruited from intensive care or high dependency hospital units were excluded. We screened studies using systematic review software and extracted data from published reports. Primary outcomes were measures of morbidity (prevalence or incidence) and mortality (standardised mortality ratios [SMRs] and mortality rates). Summary estimates were calculated using a random effects model.

**Findings:**

Our search identified 7946 articles, of which 337 studies were included for analysis. All-cause standardised mortality ratios were significantly increased in 91 (99%) of 92 extracted datapoints and were 11·86 (95% CI 10·42–13·30; *I*^2^=94·1%) in female individuals and 7·88 (7·03–8·74; *I*^2^=99·1%) in men. Summary SMR estimates for the International Classification of Diseases disease categories with two or more included datapoints were highest for deaths due to injury, poisoning, and other external causes, in both men (7·89; 95% CI 6·40–9·37; *I*^2^=98·1%) and women (18·72; 13·73–23·71; *I*^2^=91·5%). Disease prevalence was consistently raised across the following categories: infections (eg, highest reported was 90% for hepatitis C, 67 [65%] of 103 individuals for hepatitis B, and 133 [51%] of 263 individuals for latent tuberculosis infection), mental health (eg, highest reported was 9 [4%] of 227 individuals for schizophrenia), cardiovascular conditions (eg, highest reported was 32 [13%] of 247 individuals for coronary heart disease), and respiratory conditions (eg, highest reported was 9 [26%] of 35 individuals for asthma).

**Interpretation:**

Our study shows that homeless populations, individuals with substance use disorders, sex workers, and imprisoned individuals experience extreme health inequities across a wide range of health conditions, with the relative effect of exclusion being greater in female individuals than male individuals. The high heterogeneity between studies should be explored further using improved data collection in population subgroups. The extreme health inequity identified demands intensive cross-sectoral policy and service action to prevent exclusion and improve health outcomes in individuals who are already marginalised.

**Funding:**

Wellcome Trust, National Institute for Health Research, NHS England, NHS Research Scotland Scottish Senior Clinical Fellowship, Medical Research Council, Chief Scientist Office, and the Central and North West London NHS Trust.

## Introduction

Inclusion health is a research, service, and policy agenda that aims to prevent and redress health and social inequities among people in extremely poor health due to poverty, marginalisation, and multimorbidity.^1^ The association between socioeconomic status and health outcomes is well established. However, these commonly observed social gradients in health do not capture the full extent of health inequities for individuals who experience considerable social exclusion.

Previous research has described the high prevalence of substance use disorders in homeless populations,^2^ prisoners,^3^ and sex workers,^4^ and the increased prevalence of homelessness in prisoners^5^ and sex workers.^6^ These marginalised populations have common intersecting characteristics and adverse life experiences that lead to considerable social exclusion, making them powerful determinants of marginalisation in high-income settings.^7^

**Research in context**

**Evidence before this study**

A comprehensive body of research exists on the health effect of inequity, much of which focuses on disparities in morbidity and mortality, and is based on common measures of socioeconomic status, such as neighbourhood deprivation and occupational class. A consistent association has been found between ill health and increasing levels of social deprivation, which has underpinned a broad range of social policies and public health initiatives. Such analyses cannot adequately assess the extent of health inequity faced by individuals who experience considerable social exclusion. In preparation for this Review, we searched the Cochrane Library, MEDLINE, and Embase databases for articles published between Jan 1, 2005, and Sept 30, 2013. We searched for systematic reviews, meta-analyses, cohort studies, and cross-sectional studies containing morbidity and mortality outcomes for the four inclusion health populations of interest (substance use disorders, homeless populations, prisoners, and sex workers). We only included full-text articles published in English. Full search terms are listed in the appendix. The studies identified described the highly overlapping nature of inclusion health populations, the increased risk factors for disease, and poor mortality outcomes compared with the general population. Previous systematic reviews have analysed health outcomes of individual inclusion health populations, but none have examined the populations together.

**Added value of this study**

Our systematic review and meta-analysis provides the first comprehensive examination to date of morbidity and mortality outcomes across a range of inclusion health populations. We found that the extent of the health inequity seen in our inclusion health populations greatly exceeded that previously observed between populations with high and low socioeconomic status and was consistent across inclusion health populations. Mortality rates are extremely high across the International Classification of Diseases, tenth revision disease categories in inclusion health populations, and our review is the first to show that relative risks are consistently higher in female than male individuals.

**Implications of all the available evidence**

The extreme burden of disease experienced by inclusion health populations demands a cross-sectoral response to prevent considerable social exclusion and an improvement in services that work with these populations. Our analyses focused on relative measures of mortality and therefore future work should examine absolute measures in greater detail. Inclusion health populations are often invisible within routine health data. This limitation can be addressed by modifying the instruments used to collect such data or through data linkage studies. Services that provide for inclusion health populations should aim to deliver health and social services for overlapping marginalised groups to tackle the poor health outcomes found in this study. These services should also have a greater focus on prevention and management of more common conditions in addition to those traditionally considered high risk for inclusion health groups.

When considered separately, marginalised populations have been shown to have high all-cause mortality.^8^, ^9^, ^10^ However, despite the considerable overlap in risk factors and the substantially increased mortality observed in these populations, no previous review has examined the outcomes of these groups together.

No universally agreed theoretical framework exists to describe inclusion health. In this Article, we build on existing social exclusion theory and consider the so-called linked and cumulative factors and processes that confound individual and group capacity for hope, opportunity, reciprocity, and participation.^11^ Our analysis is also informed by an intersectionality perspective, which focuses on how social characteristics combine to have an effect on health.^2^, ^12^

Our systematic review therefore aims to examine mortality and morbidity in homeless populations, prisoners, sex workers, and individuals with substance use disorders, who experience considerable exclusion.

## Methods

### Search strategy and selection criteria

For this systematic review and meta-analysis, we searched the Cochrane Library, MEDLINE, and Embase for articles published between Jan 1, 2005, and Oct 1, 2015. Full search terms are provided in the appendix. We searched for articles about the populations of interest (homeless individuals, prisoners, sex workers, and individuals with substance use disorders, excluding cannabis and alcohol use) from systematic reviews, meta-analyses, interventional studies, and observational studies that had morbidity and mortality outcomes. We included studies identified from references of included articles. We only included full-text articles published in English that were done in high-income countries (classified according to the World Bank classification^13^). We excluded studies with only perinatal outcomes and did not include data on perinatal outcomes from studies that otherwise met our inclusion criteria. We excluded articles that limited the study population to individuals with a specific health condition and studies that recruited participants exclusively from intensive care or high dependency hospital units.

We recognise that social exclusion has a major effect on health in other social groups, including Gypsies and Travellers, migrants, ethnic minorities, indigenous communities, and sexual and gender minorities. Although these groups experience social exclusion in many high-income settings, they were considered beyond the scope of this systematic review.

### Data analysis

RWA screened titles, abstracts, and full texts using Covidence systematic review software. All authors contributed to data extraction, and data were double-checked by a second researcher (RWA, EJT, GH, or SVK).

Extracted items included study design, year or years of study, country, number of participants, primary outcomes, and summary descriptions of the study population. We tried to contact authors if we were unable to locate papers or required additional information about the data or study.

We attempted to identify and exclude duplicate data from research studies presented in separate publications. For cases in which we identified multiple studies with duplicated or overlapping data (by population, time, place, and outcome) we selected the study with the largest or most representative sample size, and when these were also similar, we present the most recent study. We followed the PRISMA reporting guidelines in the presentation of our manuscript. A review protocol was not published before this review was done.

Outcomes included were measures of morbidity and mortality for conditions defined in the International Classification of Diseases, tenth revision (ICD-10). Outcomes were reported using a variety of measures. To ensure maximum comparability across studies for mortality outcomes, we extracted, in order of preference, the first of the following measures: standardised mortality ratio (SMR), hazard ratio, mortality rate ratio, or crude mortality rate. For consistency with most studies included in this Article, we have not multiplied SMRs by 100. In our results, a value of 1 equates to no difference between the expected and observed mortality rate. For morbidity outcomes, we extracted, in order of preference, the first of the following measures: prevalence, incidence, prevalence risk ratio, incidence rate ratio, prevalence odds ratio, or incidence odds ratio. When available, we used data in which the comparison group was a socially deprived population or measures were adjusted for area-based or income-based deprivation.

A link to all extracted data is included in the appendix. For the quantitative findings analysed in this study, we focused the synthesis on SMRs. SMRs for all-cause mortality and by ICD-10 disease category were summarised in forest plots. We anticipated high levels of heterogeneity, and therefore did summary estimates with random effects models using Stata version 13. We used the *I*^2^ statistic to indicate the proportion of total variation in study estimates due to heterogeneity.^14^ We explored potential sources of heterogeneity by stratifying the analyses by country and by inclusion health population group.

### Role of the funding source

The funders of the study had no role in study design, data collection, data analysis, data interpretation, writing of the report, or the decision to submit the paper for publication. All authors had full access to all the data in the study and had final responsibility for the decision to submit for publication.

## Results

**Figure 1:**
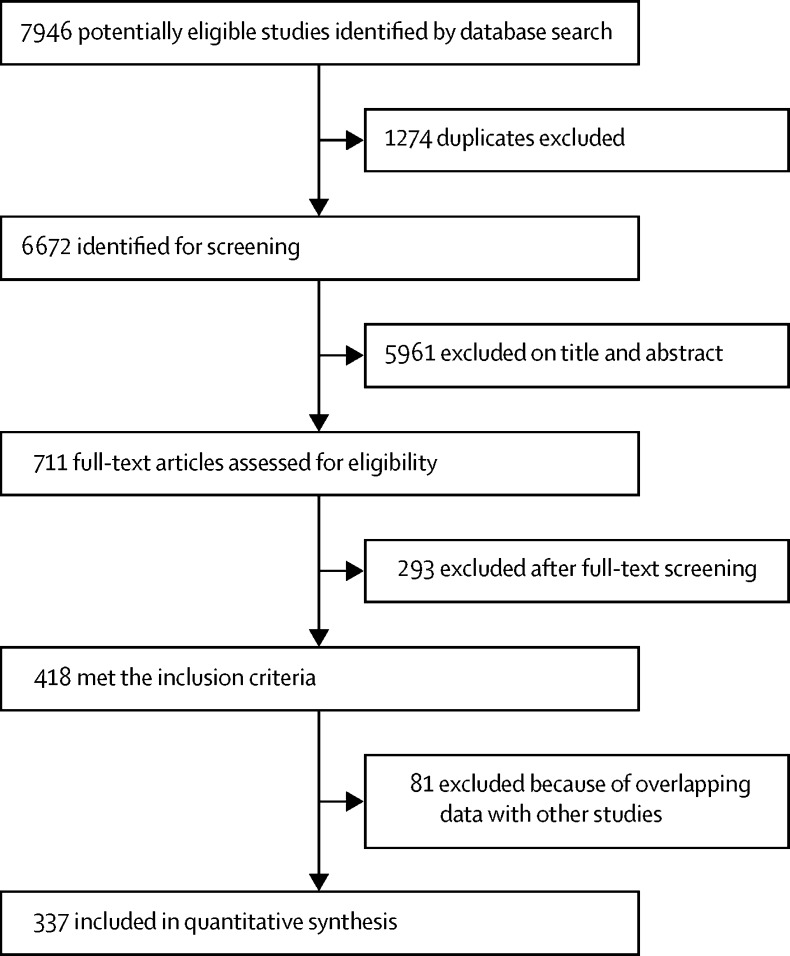
Study selection

We identified 7946 articles, of which 1274 were duplicates (figure 1). Of the 711 full-text articles retrieved, 418 met the inclusion criteria. We excluded a further 81 articles because of overlapping data. A total of 337 studies were included in this Article, which included 2835 datapoints (ie, effect estimates for a unique population) after the removal of 384 duplicates.

The studies were from 38 countries (appendix). The USA contributed 698 datapoints, Australia contributed 460, Sweden contributed 309, Canada contributed 257, and the UK contributed 234. Populations with substance use disorders were the most studied subgroup, accounting for 1193 (42·1%) of 2835 datapoints, followed by prisoners (769 [27·1%]), homeless populations (754 [26·6%]), and sex workers (119 [4·2%]).

**Figure 2:**
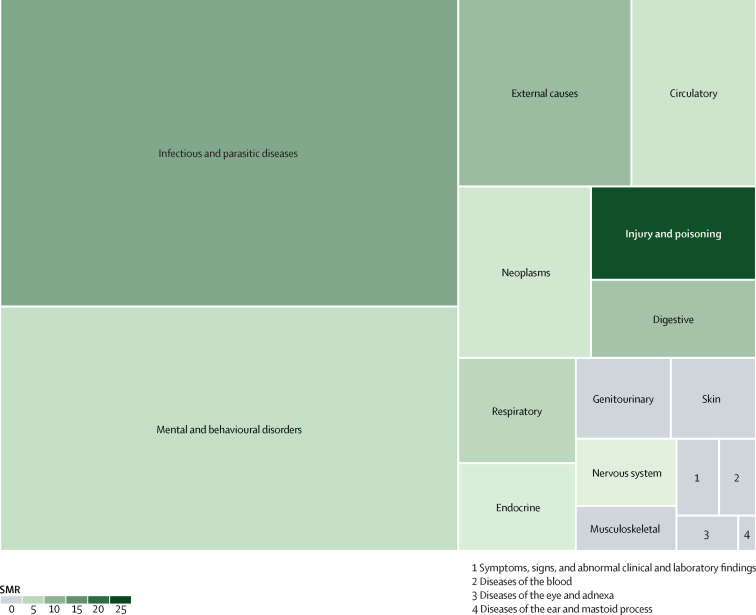
Treemap summarising the amount of available data grouped according to the ICD-10 disease categories and summary estimates of SMRs Box sizes indicate the total number of datapoints included in this Article. SMRs used are summary estimates for the ICD-10 disease categories for both sexes combined. Grey boxes (SMR of 0) indicate that none of the studies included in this Article reported SMR for both sexes combined. ICD-10= International Classification of Diseases, tenth revision. SMR=standardised mortality ratio.

Infectious diseases and mental and behavioural disorders were the two most studied ICD-10 categories with infectious diseases accounting for 898 (31·6%) of 2835 datapoints, and mental and behavioural disorders accounting for 715 (25·2%) datapoints (figure 2, appendix p 4). Injury and poisoning only accounted for 98 (3·4%) of all extracted datapoints.

**Figure 3:**
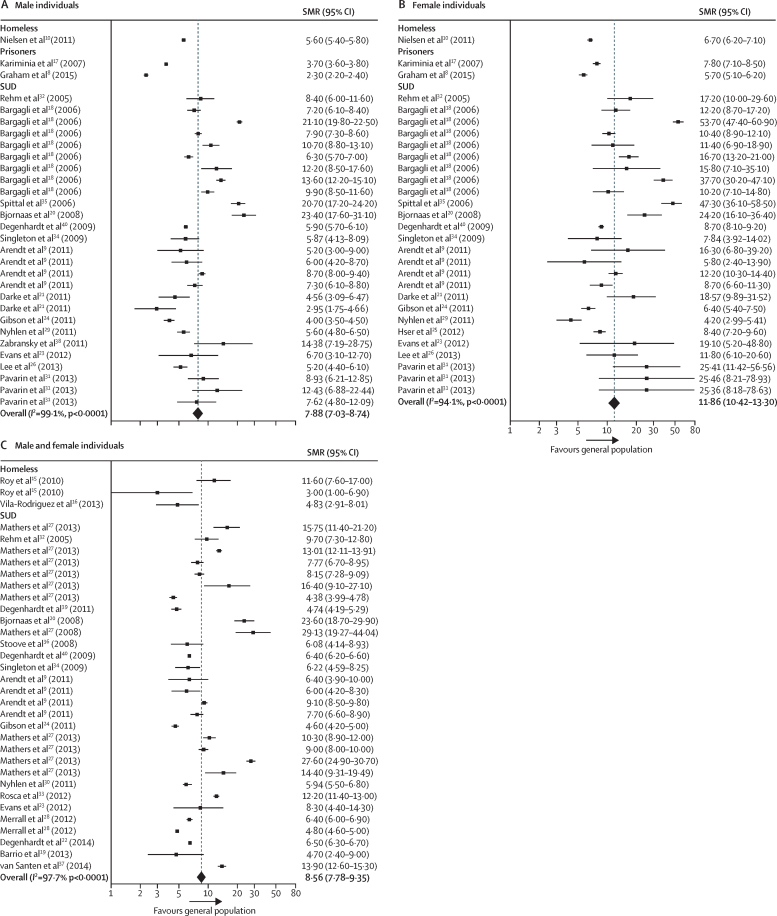
Forest plots of SMRs for all-cause mortality Data are presented for male individuals (A), female individuals (B), and overall (C). Weights were assigned by random effects analysis. Several studies contribute multiple rows of data because different populations with substance use disorders were studied,^9^, ^31^ because different countries were included,^18^ or because different time periods were studied.^28^ SMR=standardised mortality ratio. SUD=substance use disorder.

Our all-cause meta-analyses focused on SMRs and included 29 studies,^8^, ^9^, ^10^, ^15^, ^16^, ^17^, ^18^, ^19^, ^20^, ^21^, ^22^, ^23^, ^24^, ^25^, ^26^, ^27^, ^28^, ^29^, ^30^, ^31^, ^32^, ^33^, ^34^, ^35^, ^36^, ^37^, ^38^, ^39^, ^40^ which contributed 92 datapoints (table, figure 3, appendix). 91 (99%) of the 92 all-cause SMRs were increased and overall we estimated that summary all-cause SMRs were higher in female individuals (11·86 [95% CI 10·42–13·30]; *I*^2^=94·1%; figure 3) than male individuals (7·88 [7·03–8·74]; *I*^2^=99·1%; figure 3). We provide summary estimates of SMRs; however, the *I*^2^ statistic indicated that data were heterogeneous in many of our analyses and therefore these summary measures must be interpreted with appropriate caution. Heterogeneity was not substantially reduced when analyses were stratified by population subgroup (appendix). Insufficient data were available to do subgroup analyses by country.

**Table:** Studies included in the standardised all-cause mortality ratio meta-analyses

	**Study years**	**Country**	**Participants (n)**	**Population description**
**Homeless people**				
Nielsen et al^10^	1999–2009	Denmark	32 711	Women aged 16 years or older with at least one contact with a homeless shelter
Roy et al^15^	1995–2001	Canada	829	Individuals aged 14–25 years with unstable housing
Vila-Rodriguez et al^16^	2008–11	Canada	293	Prospective community sample of adults living in single-room occupancy hotel
**Prisoners**				
Graham et al^8^	1996–2007	UK	76 627	Male individuals imprisoned for the first time between 1996 and 2007
Kariminia et al^17^	1988–2002	Australia	85 203	All adults who had been in full-time custody
**Individuals with substance use disorders**				
Arendt et al^9^	1996–2006	Denmark	20 581	People receiving treatment in specialist institutions for substance use disorder, who reported cocaine as their primary substance
Bargagli et al^18^	1996–2002	Netherlands	2575	Male opiate users aged 15–69 years entering treatment
Barrio et al^19^	2004–06	Spain	714	Regular cocaine users recruited from drug scenes and non-treatment settings
Bjornaas et al^20^	1980–2000	Norway	185	Individuals with opioid addiction admitted to hospital because of self-poisoning
Darke et al^21^	2001–09	Australia	615	Opioid users
Degenhardt et al^22^	1985–2005	Australia	43 789	People who are opioid-dependent treated with opioid substitution therapy
Evans et al^23^	2005–07	USA	644	Injecting drug users younger than 30 years
Gibson et al^24^	1980–2006	Australia	2489	Opioid users
Hser et al^25^	2000–02	USA	4447	Women who were admitted to drug treatment programmes
Lee et al^26^	2006–08	Taiwan	10 842	Heroin users attending opioid substitution therapy
Mathers et al^27^	1980–99	Denmark	101	People who injected opioids and other drugs
Merrall et al^28^	1996–2006	UK	69 456	People in contact with drug treatment services
Nyhlen et al^29^, ^30^	1970–2006	Sweden	561	Substance abusers admitted for inpatient detoxification
Pavarin et al^31^	1988–2012	Italy	471	Individuals who had visited a public treatment centre for problems due to cocaine use
Rehm et al^32^	1994–2000	Switzerland	6281	Participants in heroin-assisted treatment
Rosca et al^33^	1999–2008	Israel	9818	Patients who had ever been treated or were currently in treatment in methadone maintenance treatment clinics
Singleton et al^34^	1997–2002	Czech Republic	3039	Drug users admitted to hospital for drug-related problems
Spittal et al^35^	1996–2002	Canada	520	Injecting drug users recruited through self-referral and street outreach
Stoove et al^36^	1990–2006	Australia	220	Injecting drug users recruited from the community
van Santen et al^37^	1985–2012	Netherlands	1254	Individuals recruited from local methadone outposts, a sexually transmitted diseases clinic, and by word of mouth
Zabransky et al^38^	1996–2008	Czech Republic	151	Injecting drug users aged 15–18 years
Degenhardt et al^39^	1996–2004	Canada	717	People who injected cocaine daily
Degenhardt et al^40^	1985–2006	Australia	42 676	Opioid users

Summary SMRs were higher in female individuals than in male individuals for mortality in each of the ICD-10 categories (appendix pp 6–7). In some ICD-10 categories, the summary SMRs for both sexes combined did not fall between the male and female estimates because the meta-analyses used data from different studies (rather than the estimate for both sexes combined being drawn from the male and female populations).

We identified 201 papers reporting outcomes for infectious and parasitic diseases. Summary estimates of SMRs for infectious diseases were increased in male individuals (2·83 [95% CI 1·61–4·05]; *I*^2^=65·4%; appendix p 6) and female individuals (5·58 [1·46–9·70]; *I*^2^=60·0%; appendix p 6) and both sexes combined (11·43 [6·91–15·94; *I*^2^=97·0%; appendix p 6). Disease prevalence was high but heterogeneous and ranged from 0%^41^ to 54% for HIV infection,^42^ from less than 0·1%^43^ to 90%^42^ for hepatitis C, from 2% (two of 119)^44^ to 65% (67 of 103)^45^ for hepatitis B, and from 1% (one of 82)^46^ to 51% (133 of 263)^47^ for latent tuberculosis infection.

Summary estimates of SMRs for injury, poisoning, and other external causes were the highest across all of the ICD-10 categories, in male individuals (7·89 [95% CI 6·40–9·37]; *I*^2^=98·1%; appendix p 7), female individuals (18·72 [13·73–23·71]; *I*^2^=91·5%; appendix p 7), and both sexes combined (23·53 [15·34–31·71]; *I*^2^=99·6%; appendix p 7). However, these categories only accounted for 98 (3%) of 2835 extracted datapoints. Summary SMR estimates were also increased for external causes of morbidity and mortality in male individuals (6·52 [95% CI 5·54–7·51; *I*^2^=97·4%; appendix p 7), female individuals (13·15 [9·87–16·43]; *I*^2^=93·7%; appendix p 7), and both sexes combined (8·50 [6·89–10·10]; *I*^2^=97·5%; appendix p 7). No data from studies that included sex workers were used in any of the SMR estimates for injuries or external causes.

SMRs for mental and behavioural disorders for male individuals and female individuals were exclusively from prison populations and data for both sexes combined were from populations with substance use disorders only. Only two studies included data on male individuals, one study on female individuals, and two studies on both sexes combined. Prevalence of major depression in inclusion health populations ranged from 3% (one of 38 individuals)^48^ in the month before assessment to a 53% (25 of 47 individuals)^49^ lifetime prevalence. Prevalence of schizophrenia ranged from 0·9% (212 of 23 530 individuals; we estimated the numerator on the basis of data in the original article)^50^ to 4% (nine of 227 individuals),^51^ and from 0% (none of 53 individuals)^49^ to 45% (221 of 495 individuals; numerator estimated)^52^ for bipolar disorder.

Summary estimates of SMRs for neoplasms were increased in male individuals (1·61 [95% CI 1·30–1·92]; *I*^2^=88·7%; appendix p 6), female individuals (1·91 [1·33–2·49]; *I*^2^=62·8%; appendix p 6), and both sexes combined (2·20 [1·61–2·79]; *I*^2^=90·6%; appendix p 6). Only 44 studies reported cardiovascular outcomes, accounting for 149 (5%) of 2835 datapoints extracted for this Article. Summary SMRs for diseases of the circulatory system were increased in male individuals (2·44 [95% CI 1·48–3·41]; *I*^2^=94·5%; appendix p 6), female individuals (3·13 [1·75–4·52]; *I*^2^=51·5%; appendix p 6), and both sexes combined (2·91 [2·04–3·77]; *I*^2^=85·8%; appendix p 6). The prevalence of coronary artery disease was 13% (32 of 247 individuals).^53^ Standardised mortality ratios for respiratory diseases were only reported for populations with substance use disorders and prison populations, ranging from 1·8 (95% CI 1·5–2·1)^8^ in male Scottish prisoners to 7·9 (5·1–11·8) in populations with substance use disorders in Australia.^22^ The prevalence of asthma ranged from 5·0% (10 525 of 210 501 individuals; numerator estimated)^54^ to 26% (nine of 35 individuals).^55^ Summary SMRs for gastrointestinal conditions included only data from prison populations and populations of individuals with substance use disorders, and were higher in female individuals (7·89 [95% CI 5·81–9·97]; *I*^2^=66·1%; appendix p 6) than male individuals (3·37 [2·58–4·15]; *I*^2^=93·1%; appendix p 6).

## Discussion

The excess mortality associated with considerable social exclusion is extreme. We found all-cause mortality SMRs of 7·9 in male individuals and 11·9 in female individuals. By comparison, mortality rates for individuals aged 15–64 years in the most deprived areas of England and Wales are 2·8 times higher than those in the least deprived areas for male individuals and 2·1 times higher for female individuals.^56^ The relative excesses were greatest for injury, poisoning, and external causes, but extend across almost all health conditions and across the inclusion health populations that we studied.

The available body of evidence is largest for infectious diseases, with a substantial amount of existing research on morbidity associated with mental and behavioural disorders. By contrast, evidence on non-communicable diseases and injury, poisoning, and external causes is scarce despite these causes having the highest SMRs across ICD-10 categories in our study. SMRs across disease categories were consistently higher in female than male individuals. Of the four inclusion health populations considered, sex workers were the least well investigated, which should be addressed as a matter of priority in future research.

Our study comprehensively describes for the first time, to our knowledge, the relative mortality and morbidity burden in selected inclusion health populations. We have reviewed the existing literature in this area using a comprehensive search strategy to identify the balance of evidence available to inform policy making around inclusion health. Data were extracted and reviewed by a second author to reduce the likelihood of errors. Our approach enabled the identification of relative gaps in both categories of disease and inclusion health categories. Our analysis was informed by an intersectionality perspective, which focuses on how social characteristics in combination affect health.^7^, ^57^ We have therefore specifically investigated how the health consequences of exclusion might vary as a result of other socially influenced characteristics, with differences between sexes being particularly noteworthy.

However, several limitations should be considered. Caution must be taken when interpreting the summary estimates because of the heterogeneity of studies. The absence of internationally agreed definitions of inclusion health groups is likely to explain some of this variation. Similarly, comparison groups varied, with some studies using the general population and others using groups living in socially deprived areas. Studies also varied according to the extent of adjustment for social deprivation and other risk factors. We used a random-effects method and noted the recommendations^58^ that meta-analyses should be pursued whenever possible, acknowledging heterogeneity. We limited our search to articles published from 2005 onwards and therefore we have not examined longer-term trends. Furthermore, for pragmatic reasons, we were unable to investigate other health inclusion groups and believe that further work is needed to describe their health experiences.

We found that the SMRs were consistently higher for female than male individuals. Because general population mortality rates are lower in female individuals than male individuals for most conditions, this result does not necessarily indicate that outcomes were worse in female inclusion health groups than in male groups. These results might reflect an increased vulnerability of women in inclusion health populations or different risk distributions among female individuals and male individuals in inclusion health groups. SMRs are a relative measure, and the lower (but still greater than 1) SMRs for more common diseases such as cardiovascular disease and cancer than for other conditions might underplay the number of excess cases of mortality that occurred as a result of these conditions. Conversely, high SMRs might not indicate a large number of excess deaths if the condition is rare. Further work should report absolute as well as relative measures of mortality.

These extreme inequities demand an intensive cross-sectoral policy and service response to prevent exclusion and improve health outcomes. An accompanying Review,^1^ published in *The Lancet* outlines interventions that respond to these increases in morbidity and mortality.

Determining the burden of disease remains challenging in inclusion health populations because membership of such populations is not recorded in most vital registration and health information systems. Deaths and health service use in excluded populations are therefore largely invisible and neglected aspects of routine statistics. By contrast, the availability of area-based measures of social deprivation across high-income countries has allowed the impact of less extreme social inequalities to be measured at the major population level. The outcomes of these measurements have supported extensive cross-sectoral policy initiatives to address these inequities.^59^ Better routine data is also needed to drive the policy response to the inclusion health agenda.

Two broad potential approaches are available to address this problem. First, health services could routinely record membership of health inclusion groups. This would require agreed definitions of each group. Individuals responsible for recording data would need guidance to help them ascertain membership and avoid reinforcement of stigma.^60^ The feasibility of this approach outside of specialist services remains unclear. Alternatively, and more feasibly in the short term, data linkage methods could be used to match data from services that work with inclusion health groups, with vital registration data, electronic health records, and existing disease surveillance systems.^61^ Data linkage has been the primary method used to estimate SMRs in the studies reported in this Article. These linked datasets would facilitate systematic estimates of mortality and morbidity over time and help to measure the effect of interventions.

To inform the content of this Article and the accompanying Review^1^ we held an engagement workshop with 16 people with experience of homelessness and social exclusion. We asked this group about their views on collecting operational data with ethical and appropriate research governance approvals, but without specific individual level consent. Although this sample was only small (and we acknowledge that people who face exclusion and are willing to attend a workshop might differ from those who do not), acceptability of collection of this sort of data was extremely high. 13 (100%) of 13 participants were happy for homeless hostel records to be collected, eight (73%) of 11 agreed to the collection of criminal records, eight (62%) of 13 to health records, and 11 (85%) of 13 to these records being linked together.

A vertical approach to tackling inclusion health (ie, one that focuses on specific diseases or specific risk groups) can overlook multimorbidity and the social issues faced by excluded populations.^62^ This approach can result in inefficiencies and missed opportunities for prevention, early diagnosis, and management, and missed opportunities for mitigation of social risk factors. The emerging field of inclusion health should advocate for and deliver joined up health and social services for overlapping marginalised groups. These services should address not only diseases with extreme disparities, but also prevention and management of more common conditions with a lower relative risk but high excess mortality, such as cardiovascular disease. The ability of health and social policy to address the needs of the most marginalised populations should be a key indicator of quality. Such initiatives need to be supported by information systems that can provide data for continuing advocacy, guide service development, and monitor the health of marginalised populations over time.

Our study highlights an extreme health inequity that persists in high-income countries. An inclusion health policy response must build on the evidence regarding who is at risk and the events that trigger exclusion to highlight the social and economic benefits of sustained action to prevent social exclusion.
