# Supplementary material for: Morbidity and mortality in homeless individuals, prisoners, sex workers, and individuals with substance use disorders in high-income countries: a systematic review and meta-analysis
Source: Lancet. 2018 Jan 20;391(10117):241–50. doi: 10.1016/S0140-6736(17)31869-X (PMC5803132; doi:10.1016/S0140-6736(17)31869-X)
Supplement: Supplementary appendix [file mmc1.pdf]

THE LANCET

Supplementary appendix

This appendix formed part of the original submission and has been peer reviewed. We post it as supplied by the authors.

Supplement to: Aldridge RW, Story A, Hwang SW, et al. Morbidity and mortality in homeless individuals, prisoners, sex workers, and individuals with substance use disorders in high-income countries: a systematic review and meta-analysis. *Lancet* 2017; published online Nov 11. http://dx.doi.org/10.1016/S0140-6736(17)31869-X.

Supplementary Appendix

Medline search terms 1
Embase search terms 2
Tables 4
Figures 5
Extracted data 8

Medline search terms

1. exp substance abuse, intravenous/
2. exp substance related disorders/
3. exp vulnerable populations/
4. exp prisoners/
5. exp homeless persons/
6. exp sex workers/
7. exp drug users/
8. exp alcoholics/
9. exp prostitution/
10. Systematic review.ti.
11. Meta analysis.ti.
12. Systematic review.ab.
13. Meta analysis.ab.
14. cohort.ab.
15. cohort.ti.
16. cross sectional.ab.
17. cross sectional.ti.
18. morbidity.ab.
19. morbidity.ti.
20. mortality.ab.
21. mortality.ti.
22. death.ab.
23. death.ti.
24. incidence.ab.
25. prevalence.ab.
26. prevalence.ti.
27. suicide.ab.
28. suicide.ti.
29. hospitalisation.ab.
30. hospitalisation.ti.
31. emergency department visits.ab.
32. emergency department visits.ti.
33. Africa.ab.
34. Africa.ti.

35. editorial.ab.
36. editorial.ti.
37. commentary.ab.
38. commentary.ti.
39. case series.ab.
40. case series.ti.
41. case study.ab.
42. case study.ti.
43. 1 or 2 or 3 or 4 or 5 or 6 or 7 or 8 or 9
44. 10 or 11 or 12 or 13 or 14 or 15 or 16 or 17
45. 18 or 19 or 20 or 21 or 22 or 23 or 24 or 25 or 26 or 27 or 28 or 29 or 30 or 31 or 32
46. 33 or 34 or 35 or 36 or 37 or 38 or 39 or 40 or 41 or 42
47. 43 and 44 and 45
48. 47 not 46
49. limit 48 to (human and yr="2005 -Current")

Embase search terms

1. addiction.sh.
2. homelessness.sh.
3. alcohol abuse.sh.
4. prostitution.sh.
5. high risk behavior.sh.
6. drug abuse.sh.
7. substance abuse.sh.
8. vulnerable population.sh.
9. Systematic review.mp.
10. Meta analysis.mp.
11. cohort.mp.
12. cross sectional.mp.
13. morbidity.mp.
14. mortality.mp.
15. death.mp.
16. incidence.mp.
17. prevalence.mp.
18. suicide.mp.
19. hospitalisation.mp.
20. emergency department visits.mp.
21. 1 or 2 or 3 or 4 or 5 or 6 or 7 or 8
22. 9 or 10 or 11 or 12
23. 13 or 14 or 15 or 16 or 17 or 18 or 19 or 20
24. 21 and 22 and 23
25. Africa.mp.
26. editorial.mp.
27. commentary.mp.
28. case series.mp.
29. case study.mp.

30. 25 or 26 or 27 or 28 or 29
31. 24 not 30
32. limit 31 to (human and yr="2005 -Current")

Tables

Table S1. Number of studies and data points included in the systematic review

ICD-10 chapter	Total number of data points (% of all data points)	Number of mortality data points (% of all mortality data points)
Total	2835 (100)	336 (100)
All-cause	140 (5)	92 (27)
Infectious and parasitic diseases	898 (32)	21 (6)
Neoplasms	145 (5)	41 (12)
Blood	18 (1)	--
Endocrine	66 (2)	6 (2)
Mental and behavioural disorders	715 (25)	6 (2)
Nervous system	43 (2)	6 (2)
Eye and adenexa	14 (0)	-
Ear	4 (0)	-
Diseases of the circulatory system	149 (5)	17 (2)
Respiratory system	79 (3)	8 (2)
Digestive system	82 (3)	34 (10)
Skin	44 (2)	-
Musculoskeletal	29 (1)	-
Injury, poisoning and certain external causes	98 (3)	44 (13)
External causes	207 (7)	61 (18)

Figures

Figure S1. Geographical spread of existing data from high-income countries on homeless populations.

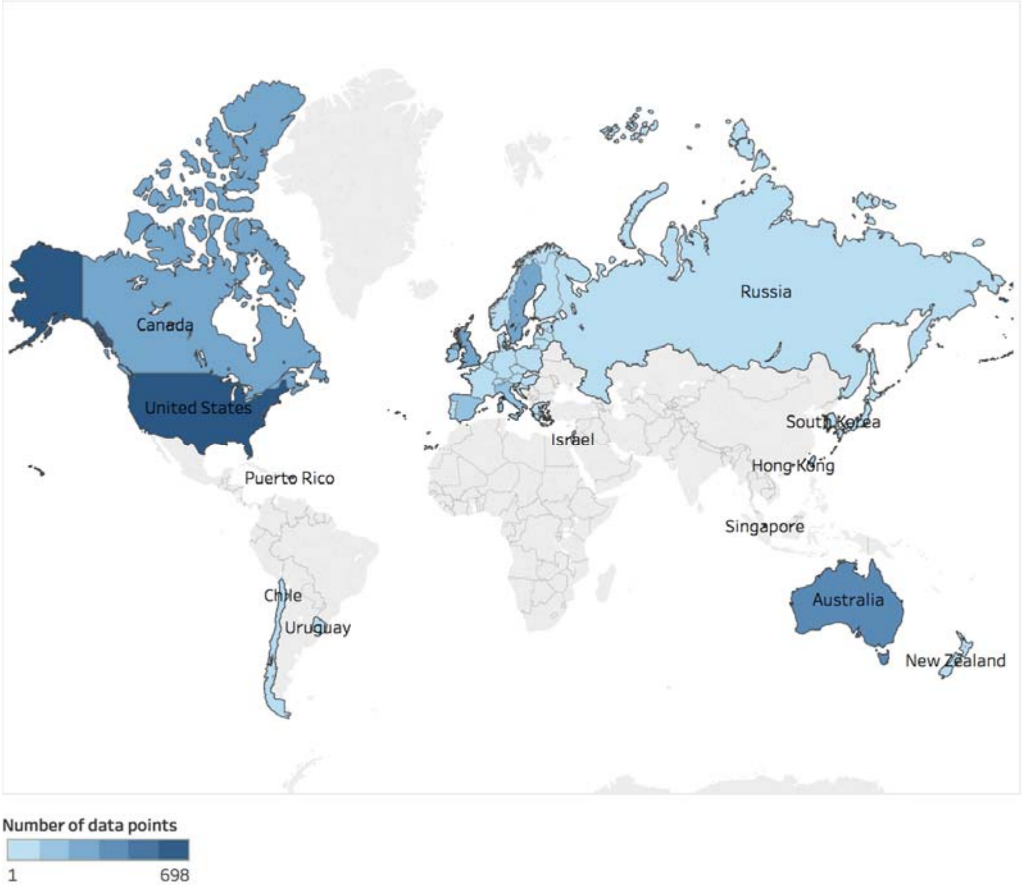

Included countries: Australia, Austria, Belgium, Canada, Chile, Croatia, Czech Republic, Denmark, Estonia, Finland, France, Germany, Greece, Hong Kong, Hungary, Ireland, Israel, Italy, Japan, Latvia, Lithuania, Luxembourg, Netherlands, New Zealand, Norway, Poland, Portugal, Puerto Rico, Russia, Singapore, South Korea, Spain, Sweden, Switzerland, Taiwan, United Kingdom, United States, Uruguay

Figure S2. Standardised Mortality Ratios by ICD-10 category (excluding those due to injury and external causes).

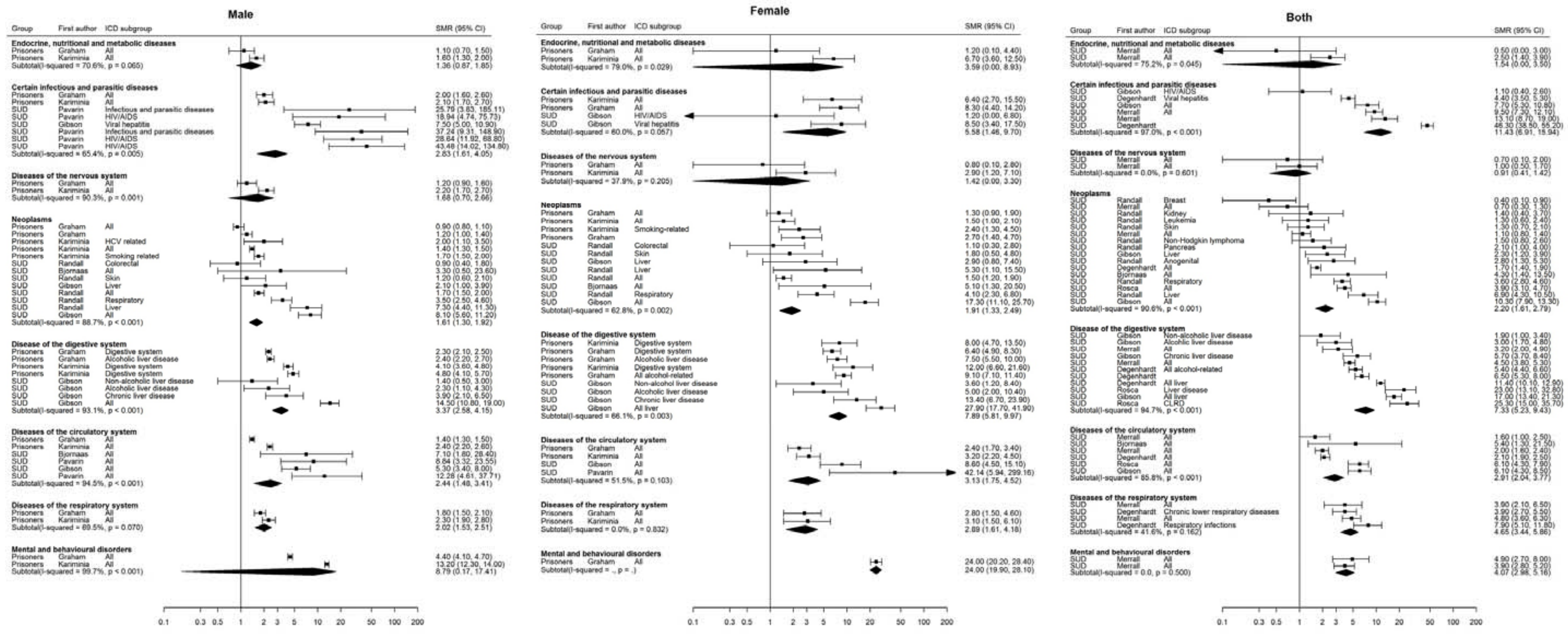

Note: Weights are from random effects analysis. Several studies contribute multiple rows of data due to different: outcomes (Graham; Pavarin; Karimina; Randall and Gibson) and time periods included (Merrall).

Figure S3. Standardised Mortality Ratios due to injury and external causes

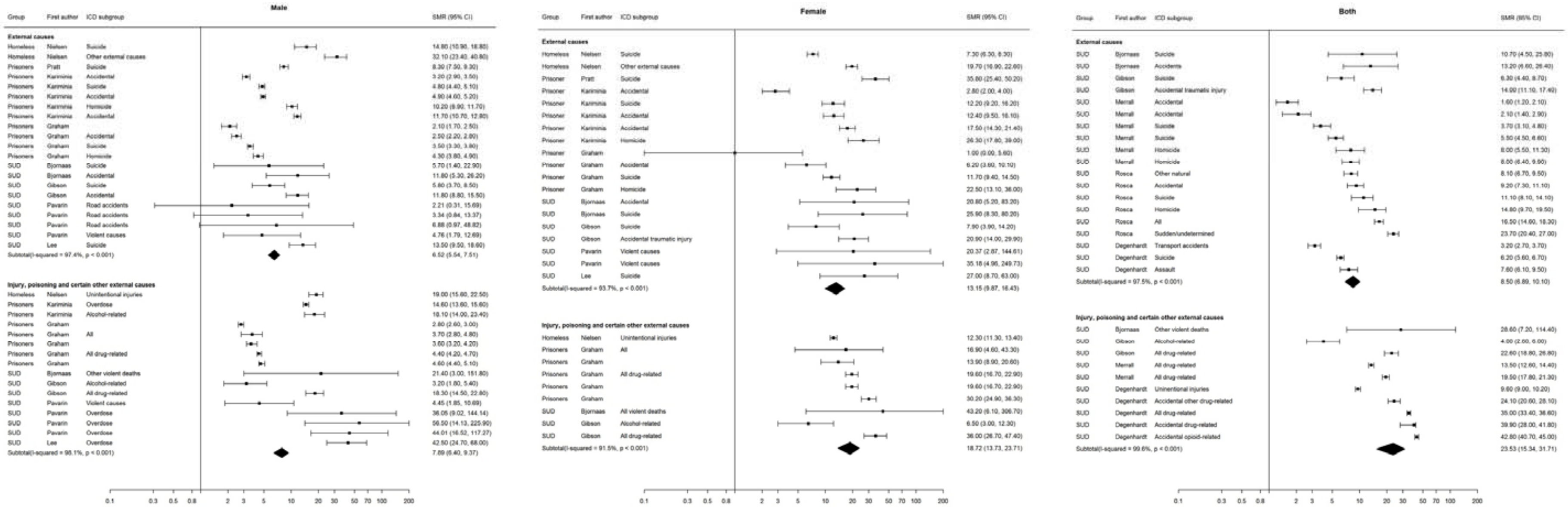

Note: Weights are from random effects analysis. Several studies contribute multiple rows of data due to different: outcomes (Nielsen; Graham; Bjornaas; Gibson; Kariminia and Degenhardt) SUD groups included (Pavarin); or time periods (Merrall).

Extracted data

Stata .dta file on all data extracted available from here:
https://1drv.ms/u/s!AjDKamSHxKsnjvlntR5Y7LQYr_Z-9w
